# T cell dynamics and response of the microbiota after gene therapy to treat X-linked severe combined immunodeficiency

**DOI:** 10.1186/s13073-018-0580-z

**Published:** 2018-09-28

**Authors:** Erik L. Clarke, A. Jesse Connell, Emmanuelle Six, Nadia A. Kadry, Arwa A. Abbas, Young Hwang, John K. Everett, Casey E. Hofstaedter, Rebecca Marsh, Myriam Armant, Judith Kelsen, Luigi D. Notarangelo, Ronald G. Collman, Salima Hacein-Bey-Abina, Donald B. Kohn, Marina Cavazzana, Alain Fischer, David A. Williams, Sung-Yun Pai, Frederic D. Bushman

**Affiliations:** 1 Department of Microbiology, University of Pennsylvania School of Medicine, 3610 Hamilton Walk, Philadelphia, PA 19104-6076 USA; 2 Imagine Institute, Paris Descartes-Sorbonne Paris Cité University, Paris, France; 3 Laboratory of Human Lymphohematopoiesis, INSERM UMR 1163, Paris, France; 4 Cincinnati Children’s Hospital Medical Center, 3333 Burnet Avenue, Cincinnati, OH 45229-3039 USA; 5 Boston Children’s Hospital, Karp 08125.3, 300 Longwood Avenue, Boston, MA 02115 USA; 6 Division of Gastroenterology, Hepatology, and Nutrition, The Children’s Hospital of Philadelphia, Philadelphia, PA USA; 7 Clinical Immunology Laboratory, Groupe Hospitalier Universitaire Paris-Sud, Hôpital Kremlin-Bicêtre, Assistance Publique-Hôpitaux de Paris, 78, r. du Général-Leclerc, 94270 Le-Kremlin-Bicêtre, France; 8 UTCBS CNRS UMR 8258, INSERM U1022, Faculté de Pharmacie de Paris, Université Paris Descartes, Sorbonne Paris Cité, Chimie Paris-Tech, 4 av. de l’observatoire, 75006 Paris, France; 9 Laboratory of Host Defenses, Laboratory of Clinical Infectious Diseases, Immune Deficiency Genetics Section, NIAID, NIH, Bethesda, MD USA; 10 Department of Medicine, University of Pennsylvania School of Medicine, 3610 Hamilton Walk, Philadelphia, PA 19104-6076 USA; 11 Biotherapy Department, Necker Children’s Hospital, Assistance Publique-Hôpitaux de Paris, Paris, France; 12 Biotherapy Clinical Investigation Center, Groupe Hospitalier Universitaire Ouest, Assistance Publique-Hôpitaux de Paris, INSERM, Paris, France; 13 Pediatric Hemato-Immunology Department, Necker Children’s Hospital, Assistance Publique-Hôpitaux de Paris, Paris, France; 14 Collège de France, Paris, France; 15 Departments of Microbiology, Immunology & Molecular Genetics; and Pediatrics, University of California, Los Angeles, USA; 16 Havard Stem Cell Institute, Harvard Medical School, Boston, MA 02115 USA

## Abstract

**Background:**

Mutation of the *IL2RG* gene results in a form of severe combined immune deficiency (SCID-X1), which has been treated successfully with hematopoietic stem cell gene therapy. SCID-X1 gene therapy results in reconstitution of the previously lacking T cell compartment, allowing analysis of the roles of T cell immunity in humans by comparing before and after gene correction.

**Methods:**

Here we interrogate T cell reconstitution using four forms of high throughput analysis. (1) Estimation of the numbers of transduced progenitor cells by monitoring unique positions of integration of the therapeutic gene transfer vector. (2) Estimation of T cell population structure by sequencing of the recombined T cell receptor (TCR) beta locus. (3) Metagenomic analysis of microbial populations in oropharyngeal, nasopharyngeal, and gut samples. (4) Metagenomic analysis of viral populations in gut samples.

**Results:**

Comparison of progenitor and mature T cell populations allowed estimation of a minimum number of cell divisions needed to generate the observed populations. Analysis of microbial populations showed the effects of immune reconstitution, including normalization of gut microbiota and clearance of viral infections. Metagenomic analysis revealed enrichment of genes for antibiotic resistance in gene-corrected subjects relative to healthy controls, likely a result of higher healthcare exposure.

**Conclusions:**

This multi-omic approach enables the characterization of multiple effects of SCID-X1 gene therapy, including T cell repertoire reconstitution, estimation of numbers of cell divisions between progenitors and daughter T cells, normalization of the microbiome, clearance of microbial pathogens, and modulations in antibiotic resistance gene levels. Together, these results quantify several aspects of the long-term efficacy of gene therapy for SCID-X1. This study includes data from ClinicalTrials.gov numbers NCT01410019, NCT01175239, and NCT01129544.

**Electronic supplementary material:**

The online version of this article (10.1186/s13073-018-0580-z) contains supplementary material, which is available to authorized users.

## Background

Several primary immunodeficiencies have been treated successfully by gene correction of hematopoietic stem cells (HSC) with integrating vectors [1–9]. This therapeutic strategy has benefited many patients and in addition provides a unique window to study mechanisms associated with immune reconstitution. In X-linked severe combined immunodeficiency (SCID-X1), the first primary immunodeficiency treated successfully by gene transfer, patients harbor mutations in the *IL2RG* gene, which encodes the common gamma chain, a component of several cytokine receptors important in T and NK cell growth and development [10–12]. Patients typically lack these cells before correction [13–15], but afterwards show robust T cell and transient NK cell reconstitution accompanied by considerable restoration of immune function [6–9]. SCID-X1 gene therapy thus provides a unique opportunity to study the consequences of T cell function in previously deficient human subjects.

In the first gene therapy trial to treat SCID-X1 (denoted here “SCIDn1”), early designs of gammaretroviral vectors were used [6–9], which were the only vector type available at the time. These vectors contained strong enhancers as part of the long terminal repeat (LTR) of the Moloney murine leukemia virus (MLV) retroviral backbone. The enhancers, along with the LTR promoter sequence, supported efficient expression of the corrective IL2RG gene and allowed successful gene correction. However, subsequent experience implicated these vectors in insertional mutagenesis, in which vector signals activated transcription of host proto-oncogenes, in some cases associated with severe adverse events [16–18].

A second trial (SCIDn2) was carried out to treat SCID-X1 using an improved self-inactivated vector in which the LTR strong enhancer sequences were deleted [19], and a promoter comprised of the short elongation factor 1 alpha (EF1a) promoter, devoid of enhancer regions, was used to express the therapeutic *IL2RG* gene. T cell numbers after correction were indistinguishable in the first and second trials. So far, no severe adverse events have been linked to insertional activation in the SCIDn2 trial after a median follow-up of 6 years for seven patients.

In this study, we used several high throughput sequence-based methods to analyze samples from the SCID-X1 trials, with the goal of probing immune mechanisms and the resulting effects on microbial communities. To assess the number and distributions of gene-corrected precursor cells producing T cells, deep sequencing of sites of vector integration was used [16–27], where each unique integration site putatively marked a distinct T cell progenitor. T cell development could be followed at a later step by using DNA sequencing to track rearrangements of gene segments encoding the T cell receptor-beta CDR3 region [28–32]. Immune cells contribute to control of the resident microbiota, so the consequences of T cell reconstitution were assessed by deep sequencing of oral, fecal, and nare samples to characterize the full microbiota using shotgun metagenomics. In a separate analysis, the viral content in fecal samples was monitored through the enrichment, purification, and sequencing of RNA- and DNA-containing viral particles.

The data support a wealth of new inferences on T cell growth and immune activity after reconstitution. For example, a minimum estimate for the numbers of cell divisions between progenitor cells and mature T cells was developed by comparing population sizes from integration site and T cell receptor (TCR) sequence data. TCR diversity could be compared for selected samples from the SCIDn1 and SCIDn2 trials, providing information on the durability of reconstitution and effects of adverse events on TCR diversity. In the microbiome data, normalization of microbial communities was documented following successful treatment in several subjects. Viral infections, several not detected clinically, could be shown to be widespread but often cleared with immune reconstitution. Thus, these data begin to outline the utility of “multi-omics” analysis of gene correction in primary immunodeficiency.

## Methods

### Human subjects

Patients were recruited as described [19, 33]. We collected the same sample types from six healthy children between the ages of 21–43 months under IRB 13-010072. We obtained sorted CD3+ T cells from three anonymous healthy adult donors above the age of 18 from the Human Immunology Core at the University of Pennsylvania. All samples were stored at − 80 °C.

### Integration site analytical methods

Integration site sequences were determined using two different methods due to changes in technology over the period of patient monitoring (details in Additional file 1: Table S2). In the first, 454/Roche pyrosequencing was used to determine integration site placement [24]. In the second, Illumina paired end sequencing was used [21, 26, 27, 34]. In both, DNA was broken using shearing or restriction enzyme cleavage, then DNA adaptors ligated on to the broken DNA ends. Nested PCR was then used to amplify from the linker to the integrated vector, and the intervening segment of human DNA sequenced. All integration site sequence analysis was be carried out in quadruplicate to minimize PCR jackpotting. All sample sets were worked up together with human DNA lacking integrated lentiviral sequences to monitor for PCR contamination, which was typically undetectable. Different linkers were used for ligation-mediated PCR for each sample in a set to block PCR cross over. All samples were bar coded on both ends of the molecule, and only those with correct bar code pairs analyzed, thereby suppressing artefactual molecules resulting from PCR recombination. A total of 31 samples were analyzed, yielding a total of 24,170 integration sites.

### TCR sequence analysis

TCR sequencing was performed on whole blood samples that had been fractionated to yield T cell (CD3+) or PBMC fractions. Genomic DNA was isolated and sequenced at Adaptive Biotechnologies using their immunoSeq protocol to determine CDR3 region sequences of the TCR-beta locus [35]. The protocol uses proprietary primer design and post-processing steps to implement controls, adjust for bias, and detect gene segments and sequence productivity. Data were analyzed using the immunoSeq Analyzer version 3.0. A total of 40 samples were analyzed, yielding a total of 32 million TCRB sequences. All statistics were performed only on the part of the clonal population for which a productive rearrangement was detected.

### Microbiome sequencing

DNA was isolated from fecal, oral, and nare samples using the following procedures: Small aliquots of fecal material (< = 1 ml) and the tips of each swab (for oral and nares swabs) were deposited into a PowerSoil bead tube. DNA was extracted using the standard MoBio PowerSoil DNA extraction protocol, with one or more blank extraction controls worked up simultaneously with each set of samples. Work spaces were decontaminated using bleach and UV irradiation. The resulting DNA from all samples and blank controls was sequenced on an Illumina HiSeq 2500 using NextSeq chemistry and standard Illumina dual barcoding for each sample.

Due to artifacts in genomic sequences for members of the Apicomplexa family, many reads from a common water contaminant (*Bradyrhizobium*) were cross-annotated as belonging to Apicomplexa. Consequently, we removed all Apicomplexa reads before further analysis due to their uncertain provenance.

### Virome analytical methods

Viral particles were isolated from a subset of the fecal samples using a protocol adapted from [36]. Fecal samples were homogenized and filtered through a 0.4-μm filter. The filtered samples were then treated with DNase and RNAseq to remove exogenous nucleic acids. Combined nucleic acids were then purified from the sample using the Qiagen UltraSens Virus Kit.

To obtain DNA viruses, we amplified viral genomes in an aliquot of the combined nucleic acids using the Illustra Genomiphi V2 DNA amplification kit. Resulting amplified DNA was quantified using PicoGreen and stored at − 20 °C. To obtain RNA viruses, we treated a separate aliquot of the combined nucleic acids with DNAse+ and then performed reverse transcription of the RNA to cDNA using the SuperScriptIII First-Strand Synthesis System from Life Technologies and second strand synthesis using Sequenase. The resulting cDNA was quantified with PicoGreen and stored at − 20 °C.

Resulting DNA was prepared and sequenced using NextSeq for library preparation and an Illumina HiSeq 2500 for sequence acquisition. The same post-processing pipeline was used, including all quality-control, host removal, and read annotation steps. For analysis, we considered only reads that fell under the virus classification and removed the following viral annotations as being reagent contamination [37, 38]: Enterobacteria phage M13, Enterobacteria phage T7, Enterobacteria phage phiX-174 sensu lato, Bacillus phage phi29, and Pseudomonas phage phi6, human herpesvirus 6 and 7, and Shamonda virus. Influenza sequences were detected, but only the HA segment was found. The HA sequence matched sequences from a previous run of the sequencing instrument, and so were excluded as carry over.

### Bioinformatic methods

To estimate population sizes of T cell progenitors from integration sites, we used Chao1, and of TCR sequence data, we used Chao2.

Sequence reads for all metagenomic samples were processed using the Sunbeam pipeline (https://github.com/sunbeam-labs/sunbeam). Reads were quality-controlled by trimming low-quality bases and adapter sequences using Trimmomatic [39], and host reads were removed using BWA [40]. The remaining reads were filtered for low-complexity sequences using dustmasker [41] and Komplexity (https://github.com/eclarke/komplexity). The reads that remained were assigned taxonomy via Kraken [42] and a custom database built on all microbial genomic sequences in RefSeq release 79 [43].

Antibiotic resistance gene levels were assessed using ShortBRED [44] and a marker gene database built from CARD [45] available on the ShortBRED website (https://bitbucket.org/biobakery/shortbred/downloads/ShortBRED_CARD_2017_markers.faa.gz). The same reads used as input to the Kraken taxonomic classifier were used as input to ShortBRED.

T cell receptor antigen recognition was queried using VDJdb [46], a database and software package that compares CDR3 regions from TCR sequencing experiments to public epitope databases. The software compared our TCRB sequences to human TCRB sequences in the database with allowances for low numbers of indels and substitutions (default parameters), and only matches with high confidence (VDJdb score > 2) were kept.

Final analysis and figure generation were performed using the R statistical software [47]. Bray-Curtis dissimilarity and other ecological metrics were calculated using the R package vegan [48]. The full code listing and post-processed data used for analysis and figures are available online at https://github.com/eclarke/scid-multiomics-paper.

## Results

### Experimental strategy

**Fig. 1:**
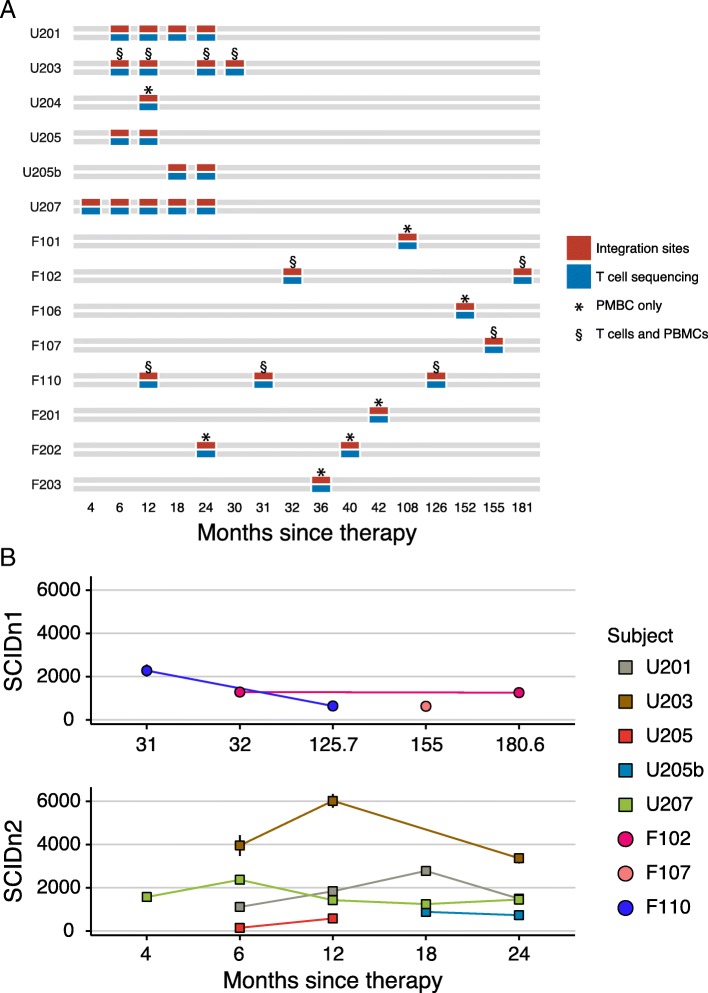
Sampling schedule for 14 SCID-X1 gene-corrected subjects studied for integration site distributions and TCRB CDR3 sequence composition. **a** Times of sample acquisition after cell infusion. **b** Population sizes of inferred progenitor cells deduced from marking with unique sites of vector integration. Integration site data is in Additional file 1: Table S2, and summaries of genes near sites of vector integration are in Additional file 2: Figure S1. The *x*-axis shows the time since cell infusion. The *y*-axis shows the population size reconstructed using Chao1 from the numbers of unique integration sites and replicate sampling. Samples are named for the site of gene correction (U indicates USA, F indicates France; the next digit indicates the trial 1 = SCIDn1, 2 = SCIDn2, and the next two digits indicate the patient number within that trial). Patients F107 and F110 suffered severe adverse events at months 68 and 33, respectively. Both recovered by the final timepoints listed

Our comparative analysis of gene-corrected progenitors and daughter T cells focused on nine patients from the SCIDn2 trial [19] and five patients from the SCIDn1 trial [8] for whom samples were available (Fig. 1a). Patient characteristics are summarized in Additional file 1: Table S1. Two of the subjects from the SCIDn1 trial developed leukemia requiring chemotherapy: F107 and F110 at 68 months and 33 months, respectively, after infusion of gene-modified progenitor cells [8, 19, 25]. The samples for these patients analyzed here are well after these adverse events, allowing assessment of the effects of leukemia and chemotherapy on progenitor cell and T cell populations. One subject in the SCIDn2 trial, U205, was transplanted twice—T cell numbers failed to reach a clinical protocol end point after the first infusion with modified cells, but succeeded the second time. Samples from the two treatment periods are designated U205 and U205b. Patient U204 was not successfully reconstituted but was rescued after allogeneic stem cell transplantation.

Vector integration sites and the T cell repertoire were monitored through regular per-protocol blood draws, followed by isolation of CD3+ populations. Sorted cells were subject to DNA extraction and then amplification of either integration sites (mostly previously reported in [8, 19, 25]; Additional file 1: Table S2) or mature TCR-beta loci (Additional file 1: Table S3; new data here), followed by sequencing. Vector integration sites mark progenitor cells capable of differentiating into mature peripheral T cells. Rearranged TCR-beta loci identify mature T cells present in the blood. Time points from the integration site analysis were chosen to match those used in the TCR analysis—characterization of additional time points for integration site distributions in SCID-X1 gene therapy was published previously [8, 18, 19, 33, 49–51].

Microbiome samples were available for six SCIDn2 patients (U201, U203, U204, U205, U207, and F201). Oral, nasal, and gut microbiota were sampled via collection of oropharyngeal swabs, nasopharyngeal swabs, and stool samples. Sampling times ranged from 4 to 181 months post infusion of corrected cells. Sample acquisition was at times limited by clinical and practical considerations.

As controls, we analyzed TCR-beta sequences from CD3+ cells from five healthy children and three healthy adults after informed consent. Healthy subject demographics are in Additional file 1: Table S1. Cross-sectional microbiome samples were collected from the same subjects and analyzed along with the SCID gene-corrected samples.

### Integration site analysis

To characterize the hematopoietic progenitor cells capable of differentiating into peripheral blood T cells, we determined the sites of vector integration in patient chromosomes. Because of the large size of the human genome, each integration site uniquely marks a precursor cell capable of additional division after integration of the vector. Summaries of genes near integration sites in the most expanded clones at each time point are in Additional file 2, Figure S1. The numbers of integration sites detected in purified T cell samples ranged widely, from as few as 62 to as many as 2009. Reconstruction of population sizes using the Chao1 estimator suggested minimal sizes of 144 to 6018 active progenitors.

For the SCIDn2 subjects, we found that the estimated total number of vector integration sites was relatively constant over the time intervals analyzed (Fig. 1b), in the range of ~ 1000 predominant clones yielding circulating cells (Additional file 1: Table S2). Numbers varied by subject, with subject U203 showing consistently higher levels, while U205, a subject in which a suboptimal number of gene-modified cells were delivered, showing consistently lower levels.

For the SCIDn1 subjects, numbers of unique sites identified varied from 263 to 682; reconstructed minimal population sizes ranged from 628 to 1287. Comparison of mean values shows no difference in the numbers of unique integration sites in SCIDn2 subjects compared to SCIDn1 subjects (*p* = 0.17, Wilcoxon rank-sum test).

Population sizes of progenitors were compared between SCIDn1 subjects who suffered adverse events and were treated with chemotherapy (F107, F110) versus those who did not (F102, F106), or versus SCIDn2 subjects. Treating time points as independent tests, no systematic differences were detected comparing within SCIDn1 subjects; a modest difference in median values was detected with a comparison of adverse events versus no adverse events (adverse event samples compared to pooled SCIDn1 and 2; *p* = 0.041, Wilcoxon rank-sum test). Thus, the data suggest that adverse events and chemotherapy may have diminished the pool size, though we note that these subjects were also at relatively long times after gene therapy, so slow loss of diversity over time is a potential alternative explanation.

### TCR-beta CDR3 analysis

**Fig. 2:**
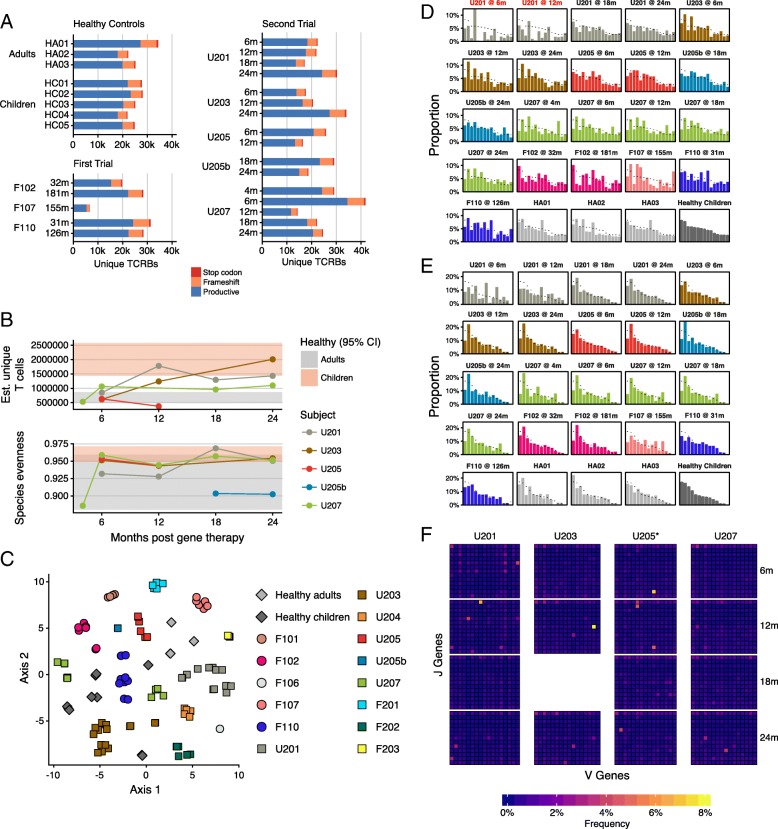
Analysis of TCRB CDR3 sequences. **a** The unique numbers of rearranged genes detected are shown. The colors indicate in frame rearrangements (blue), frameshifts (tan), and stop codons (red). **b** Richness and evenness of the inferred TCRB CDR3 populations. Patients are color coded as indicated on the right, and all replicates for each patient timepoint displayed. The ranges of healthy adults and children are shown by the light and dark gray diamonds, respectively. **c** Clustering of the samples sequenced using Bray-Curtis similarity and t-SNE. The association of patients with samples is shown by the key at the right. **d** V gene usage. The patient of origin is marked at the top of each panel. The V genes shown were the top most commonly found V genes in healthy children, ordered by prevalence; not all V genes used by each patient are shown. All panels show the same *x*-axis of V genes. Chi-squared test used to assess differences between gene distribution in patients compared to healthy children; red label indicates significant difference from healthy (*p* < 0.05). **e** J gene usage, with J genes determined by the same manner as in D. **f** Heat map summarizing the frequencies of utilization of the most common V and J pairs. Subjects studied are marked at the top. Time of sampling is shown on the right

Clonal structure of T cell populations was investigated by analyzing TCR-beta rearrangements in genomic DNA from peripheral blood CD3+ cells (Fig. 2). CDR3 region nucleotide sequences were conceptually translated into amino acid sequences, and numbers of productive rearrangements quantified (Fig. 2a). All the following analysis focused on T cell clones with productive rearrangements. For healthy adults, productive rearrangements ranged from 18,000 to 27,000 per sample. For healthy children, numbers ranged from 18,000 to 22,000 per sample. For SCIDn2, most samples were close to this range (12,000 to 33,000 per sample; no significant difference in medians). For SCIDn1, results were slightly lower, with four out of five samples between 15,000 and 23,000. The exceptional sample was from subject F107 who only showed 5,000 unique CDR3 sequences. F107 both suffered an adverse event at month 68 and was treated by chemotherapy, and demonstrated a low number of engrafted gene-modified cells at baseline. Patient F110 was also treated by chemotherapy following a serious adverse event at month 33, but was initially treated with a higher dose of gene-modified cells, potentially explaining the higher diversity in this patient.

For all the CDR3 samples studied, we could only sample a small fraction of the total CDR3 clonotypes in the subjects. To account for this, we used replicate sampling and the Chao2 population estimator to estimate a minimum bound for the true number of CDR3 clonotypes (Additional file 1: Table S3). Estimators can be sensitive to sampling effort, so our estimates represent minimum values. Focusing on samples analyzed with multiple replicates, we estimate population sizes of 26,000 to 2,600,000 CDR3 variants. The median for SCIDn2 samples was not different from that of healthy children, but the median for SCIDn1 was significantly lower than for the healthy children (*p* = 0.026; Wilcoxon rank-sum test), possibly due to the much longer follow-up time available for the SCIDn1 patients. Analysis of trends over time suggested that subjects U201, U203, and U207 repertoires became more rich over time (Fig. 2b). The one exception was the case of suboptimal reconstitution (U205), which did not show longitudinal increase.

To begin to characterize repertoire composition, we used Bray-Curtis dissimilarity and t-SNE to cluster samples (Fig. 2c). Replicates from each subject closely resembled those from the same subject at different time points, but differed from other subjects. No systematic differences were observed comparing SCIDn1 and SCIDn2, or comparing either data set to healthy controls (PERMANOVA test on Bray-Curtis dissimilarity, n.s.).

Recombination involving the most frequently used V (Fig. 2d) and J (Fig. 2e) gene segments was next quantified and compared. Usage of gene segments was quantified for healthy children and averaged, then the profile was compared to gene-corrected SCID subjects. The great majority of gene-corrected samples did not show significant differences from the distribution in healthy children, in the analysis of either V or J gene segment usage (chi-squared test). The only exception was V gene usage in early time points for SCIDn2 subject U201 (chi-squared test, *p* < 0.05). This subject was successfully corrected, so the unusual heavy use of specific V gene segments is not indicative of clinical failure. Of possible significance, U201 had an ulcer on his palate of unknown origin that became inflamed as T cells were produced, possibly skewing the repertoire. Fig. 2f shows the usage of V-J pairs within subjects, emphasizing the occasional outgrowth of expanded clones, potentially in response to antigen, followed by decrease of abundance.

TCR sequences were scanned for evidence of public epitopes in order to assess whether the subjects had formed typical responses to common antigens. However, no clear-cut link was found between putative epitopes and the microbial and viral taxa described below.

### Tracking T cell ontogeny

**Fig. 3:**
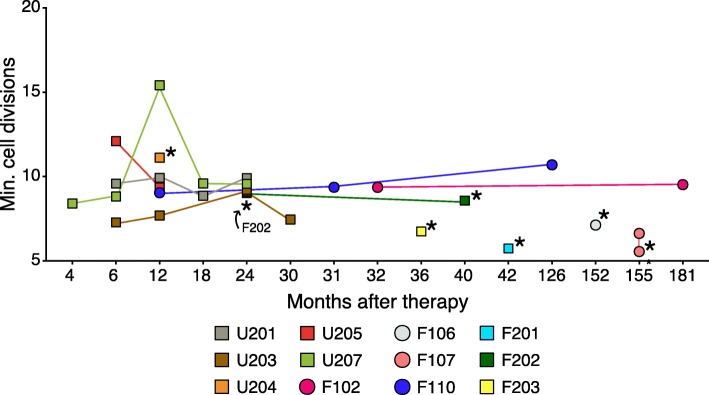
Minimum numbers of cell divisions between progenitors and daughter T cells. The *x*-axis shows time after corrected cell infusion. The *y*-axis shows the estimated number of cell divisions calculated as described in the text. The subjects studied are indicated beneath the figure as indicated by the color code. Circles indicate subjects from the second trial, and squares indicate subjects from the first. Stars next to the points indicate PBMCs were sequenced rather than sorted CDR3+ cells. Replicates were not available for U205b and so this subject was not analyzed

Comparison of the estimated lower bounds for the number of unique integration sites per sample with the number of unique TCR sequences allows estimation of the minimum number of cell divisions required to generate the TCR-beta cell population from gene-corrected precursors (Fig. 3). We calculated the minimum number of cell divisions as the base-2 logarithm of the difference between the estimated population size of unique T cells and the estimated population size of integration sites:

$$ \mathrm{CellDivisions}={\log}_2\left(\mathrm{TCRs}-\mathrm{IntSites}\right) $$

We found a relatively consistent range of minimum cell division values across patients and timepoints, with a median of 9.1 and a minimum and maximum of 5.6 and 15.4. The fraction of progenitor cells that die in the thymus is unknown, so our estimates are lower bounds for the required number of cell divisions.

### Response of the microbiome to T cell reconstitution

**Fig. 4:**
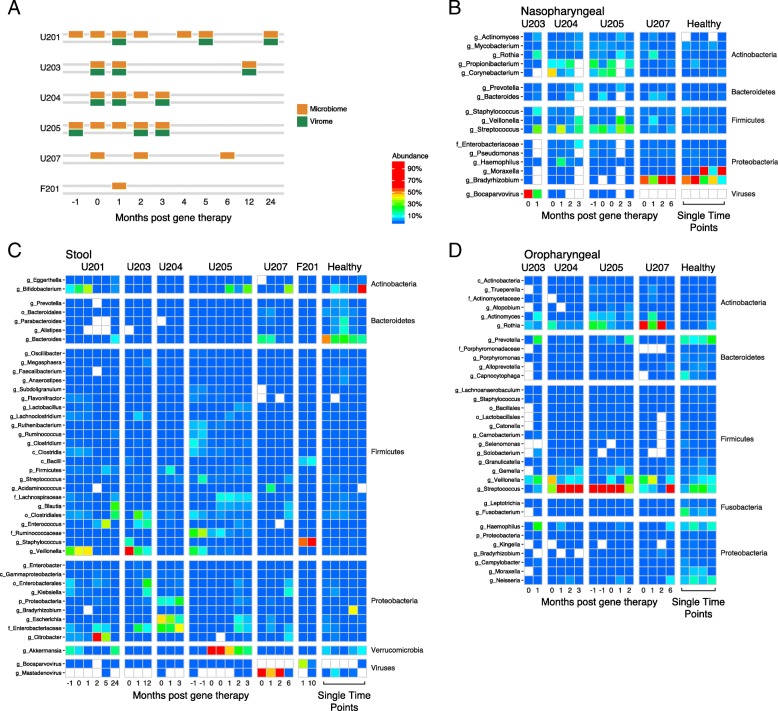
Longitudinal analysis of the microbiome during SCID-X1 gene correction. **a** Timing of sample acquisition. **b** Longitudinal analysis of the nasopharyngeal microbiome. Each column indicates a sample. Samples are grouped by subject as indicated at the top. Each row summarizes the proportions of a specific microbial taxa inferred using Kraken. Abundance is color coded as indicated to the right and reflects the number of reads assigned to that taxa as a proportion of all non-human reads. **c** As in B, but oropharyngeal samples. **d** As in B, but stool samples

Microbiota community structure was analyzed for six gene-corrected subjects (Fig. 4a) by extraction of DNA from swabs (oral and nares samples) or stool, followed by shotgun metagenomic sequencing (samples analyzed are in Table S4; summaries of clinical infections are in Table S5). Numbers of samples available per subject ranged from one to seven. The numbers of sequences acquired per sample averaged 72,000 (nasal; Fig. 4b), 8,746,000 (fecal; Fig. 4c), and 3,625,000 (oral; Fig. 4d). Sequencing reads were quality filtered as described in the “Methods” section and then assigned to microbial taxa using Kraken [42] .

The analysis of gut microbiota (Fig. 4c) emphasized the differences between gut microbiota of healthy children (right columns) versus SCID gene-corrected subjects (left columns). The healthy subjects were colonized predominantly with Bacteroides, which is typical of healthy gut, whereas the SCID subjects showed a range of major colonists. U201 was colonized mainly with Bifidobacteria, which is characteristic of healthy breast-fed babies [52]. However, the guts of subjects U203 and U205 were colonized with Veillonella at early timepoints. As Veillonella is commonly an oral bacteria, this is suggestive of abnormal colonization. Subject U204 showed high-level colonization with Enterobacteriaceae, typical of dysbiotic states. Early samples from subjects U207 and F201 showed abundant representation of Adenovirus and Bocavirus in the gut (Fig. 4c, bottom rows), comprising a significant portion of the classified taxa even though whole stool (and not virus-enriched material) was sequenced.

Oral samples (Fig. 4d) from the healthy controls were dominated by typical oral bacteria, including Prevotella, Streptococcus, Neisseria, and Haemophilus. In contrast, U204 and U205 showed high-level domination by Streptococcus, and U207 was dominated by Rothia. Viral sequences were rare in oral sample.

In nasopharyngeal samples (Fig. 4b), healthy subjects were dominated by Moraxella, Staphylococcus, and Propionibacterium. Bradyrhizobium was detected in B207 and healthy subjects, but this is a soil bacteria and a common contaminant, so we infer that detection of this lineage is artifactual. SCID subjects contained normal lineages to varying degrees, but also showed high-level colonization by Streptococcus, Corynebacterium, and others. In addition, nasopharyngeal samples from U203 were dominated by Bocavirus.

**Fig. 5:**
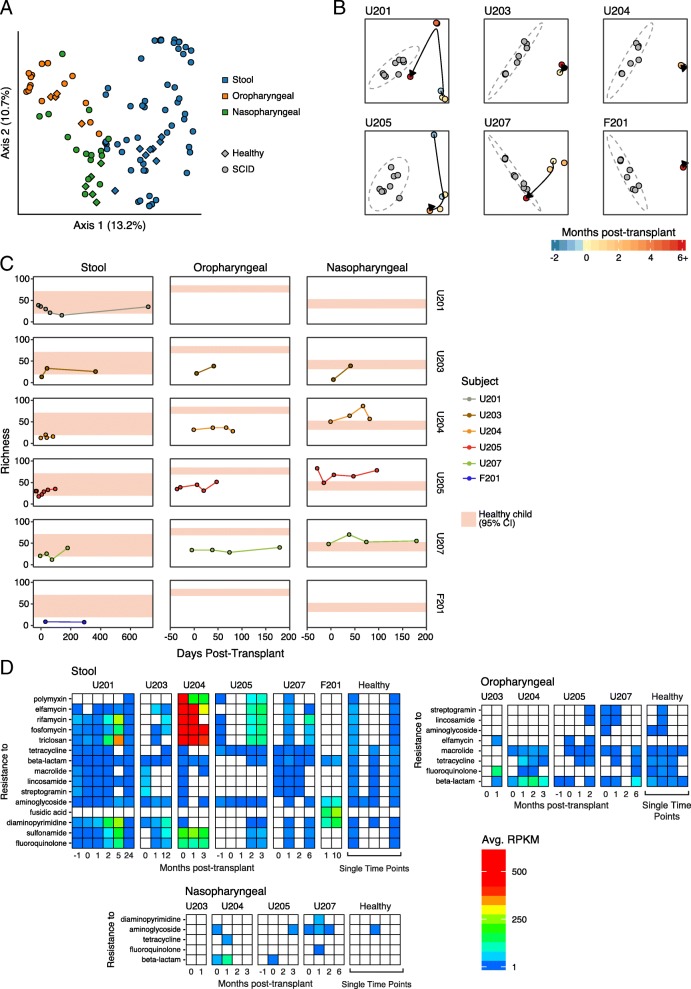
Microbiome community analysis. **a** Sample clustering using Bray-Curtis dissimilarity. Different sample types are marked by the colors, healthy versus SCID are shown by the shapes. **b** Comparison of stool samples for each patient queried to healthy controls. Samples were clustered using Bray-Curtis dissimilarity. Each panel compares one SCID subject samples (indicated at the top) to healthy control samples (shown in gray). Elapsed time is shown using the color code (bottom). **c** The longitudinal species richness of each patient’s oral, nasal, and stool samples is shown in separate panels. The 95% CI of the healthy child richness for that sample type is shown in tan. **d** Representation of selected antibiotic resistance genes in the stool samples studied. Each column indicates a metagenomic data set from the subject listed at the top. Each row summarizes the abundance of an antibiotic resistance gene class. The tiles are colored by reads per kilobase of target per million sequence reads (RPKM); the color code is to the right of the panel

We compared the full microbial compositions of the samples using Bray-Curtis dissimilarities and found that sample types clustered by body site of origin (Fig. 5a, PERMANOVA *p* value < 0.001), as expected from many studies. Nasopharyngeal swabs also clustered near the reagent controls, suggesting that low authentic microbial biomass was present in these samples and that a significant proportion of the reads were likely derived from environmental contamination. We also found that each subject clustered with themselves throughout all timepoints, reflecting consistently higher inter-subject differences than intra-subject differences (PERMANOVA *p* value < 0.001). In several patients (U201, U205, and U207), the gut microbial communities began resembling healthy children more at late times after cell infusion (Fig. 5b), potentially reflecting a combination of improving immune function and reduction in usage of antibiotics and other medications. U203 and F201, whose microbial communities remained abnormal throughout the sampling period, were on prolonged antibiotics for treatment of disseminated Bacille Calmette-Guerin after gene therapy, potentially contributing to dysbiosis.

Analysis of taxonomic richness provides insight into microbial community health, since low richness is often associated with abnormal outgrowth of pathogenic organisms. For stool, richness increased for 4/6 subjects over time, potentially indicative of improving gut health (Fig. 5c). However, of the four subjects for whom oral samples were available, all showed abnormally low richness at every time point. This finding was associated with particularly high Streptococcus colonization in multiple subjects. Nasopharyngeal microbiota showed richness comparable to healthy controls.

SCID patients were treated with a wide variety of antibiotics before and after therapy to mitigate opportunistic infections. To assess whether this treatment was associated with an increase in antibiotic resistance gene representation, we quantified antibiotic resistance genes in the stool sequence samples using ShortBRED and the CARD antibiotic resistant factor database (Fig. 5d). We saw a greater quantity of antibiotic resistance genes in SCID subjects than in healthy controls in stool (pooled across timepoints, *p* = 0.048). Particularly high levels were seen in U204, for whom gene therapy was unsuccessful. Lesser frequencies of antibiotic resistance genes were evident in the oropharyngeal and nasopharyngeal samples. For patient U201, who had a timepoint 24 months after therapy, the level of antibiotic resistance genes decreased substantially between the 5- and 24-month timepoint. At this timepoint, the patient was no longer on any medications, which suggests that antibiotic resistance gene load may have decreased after the selective antibiotic was withdrawn. This suggests that the antibiotic administration, and potentially hospitalization, increased antibiotic resistance gene proportions.

### Response of the virome

**Fig. 6:**
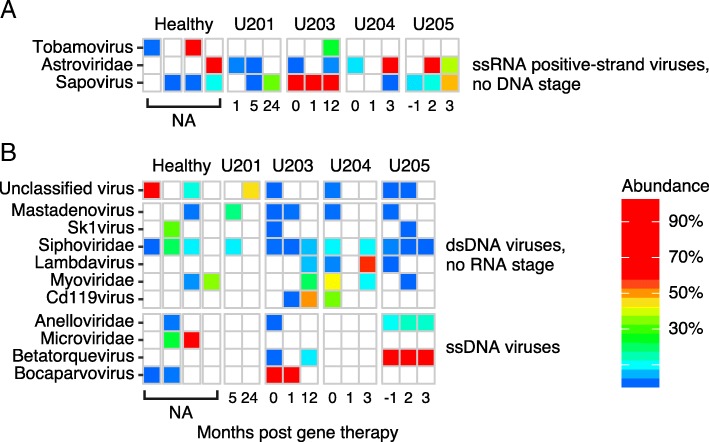
Virome analysis. **a** Heat map summarizing RNA viruses detected. Each column indicates a sample from the patient indicated at the top of the heatmap, each row indicates a type of virus. The tiles are colored according to abundance. **b** Heat map summarizing DNA viruses detected. Markings as in A

To enrich for virus detection beyond what was identified in whole stool sequencing (as in Fig. 4), viral particles were purified from stool, and DNA and RNA purified from particles and sequenced. Only patients with sufficient material were able to be virome-sequenced (patients U207 and F201 were excluded). Reads were assigned using Kraken, then extensive filtering was carried out to remove contaminants and artifacts. Figure 6 shows the remaining attributions. For RNA viruses, high-level colonization was detected with Astrovirus (U204 and U205) and Sapovirus (U203). For DNA viruses, numerous bacteriophage lineages were seen. Among DNA viruses infecting animal cells, high viral loads were seen for Adenovirus in U201, Bocavirus in U203, and Betatorquetenovirus (Anellovirus) in U205. The Adenovirus and Bocavirus infections diminished over time after successful gene correction. Betatorquetenovirus is a normal commensal in healthy individuals and persisted.

## Discussion

We present here an initial analysis of the co-development of the microbiome and immune system in patients after gene therapy for SCID. We used targeted sequencing of vector integration sites to model the number of gene-corrected progenitor cells and TCR sequencing to capture the development of the T cell repertoire following therapy. The combination of these data allowed a lower-bound approximation of the number of cell divisions required to progress from a lymphoid progenitor cell to a circulating T cell, a measurement uniquely possible due to the combined analysis. We used shotgun metagenomic sequencing in longitudinal samples to track early-term changes in the gut, oral, and nasopharyngeal microbiome accompanying therapy. In a subset of these timepoints, we purified and sequenced RNA and DNA viruses, revealing clearance of several viruses associated with immune reconstitution.

The TCR-beta CDR3 analysis provided new evidence on how SCID gene therapy patients progressed to healthier phenotypes after successful therapy. After successful reconstitution, their T cell richness, as measured by TCR-beta sequencing, consistently moved from the oligoclonal range to a polyclonal range characteristic of healthy children. Further, their V and J usage generally showed a pattern typical of a healthy and age-typical state. This is concordant with the observed results of healthy immune function in these patients and shows that sequencing metrics of the T cell repertoire correlated with clinical outcomes here. A number of patient repertoires had outgrowths of expanded clones, possibly due to response to antigens. We found lower T cell diversity in SCIDn1 patients than healthy controls, which may indicate a slow but gradual loss of diversity with time after therapy.

Comparison of the population sizes of progenitor cells, marked by unique integration sites, and T cell clones, marked by unique TCR-beta sequences, suggested that intrathymic cell expansion from progenitors to T cells involved at least nine cell divisions. In comparison with our result, a related estimate was derived from a study of a SCID-X1 patient with spontaneous reversion of a *IL2RG* point mutation, in which descendants of a single reverted progenitor cell repopulated the immunodeficient subject [53]. In that case, it was estimated that at least 10 cell divisions were required to expand the single revertant T cell progenitor to yield the observed 1000 TCR-beta clonotypes. Though we note that both methods have large and uncharacterized uncertainties, the two yield notably similar results. In the case of SCID-X1 gene therapy, if (very roughly) 1000 progenitor cells supported long-term immune reconstitution, then about 10 cell divisions (2^10^ = 1024) would be required to reach the same final population size of around 1 million distinct T cell clones with unique TCR-beta sequences. Each clone will then further expand in the periphery to allow the production of the patient final T cell pool containing around 10^9^ T lymphocytes (about 10 additional divisions). In comparison, in the revertant subject, 20 additional divisions will be required to allow the production of the final T cell pool in the periphery. That is, 10 divisions in gene therapy subjects and 20 in the revertant would produce a similar final population size. Implicit in these observations is the hypothesis that there exists a feedback mechanism that determines the final size of the T cell pool.

As with their TCR repertoires, the microbiota of several successfully treated patients changed to more resemble those of healthy children. We saw an increase in diversity and richness of the microbes in the gut, oro- and nasopharyngeal compartments, and an outgrowth of bacteria associated with healthy gut function. In some patients, we were also able to detect bacteria that may be associated with negative outcomes and antibiotic resistance, potentially as a result of extended exposure to antibiotics and hospitalization. In one patient where longer term samples were available, we documented a loss of antibiotic resistance genes at the last time point, suggesting that maintenance of the resistant population is largely reliant on the selective pressure exerted by the drug. NK cell reconstitution in SCID patients is variable, so our data emphasize the importance of T cells in regulating the microbiota versus NK cells [19, 54].

An unexpected finding was the consistently low diversity of SCID oral samples, and the high colonization with Streptococcus. It will be of interest to assess whether this is seen in other immunodeficient subjects, and target investigations of possible pathology associated with Streptococcus.

In some patients, we found viruses known to cause enteropathic conditions in humans, including Astrovirus, Bocavirus, and Adenovirus. In the case of patient U207, the adenovirus detection was corroborated with a clinical assay, and for U203, Bocavirus was detected clinically, supporting the reliability of the metagenomic detections. In each, the relative abundance of viral reads was diminished at later timepoints, likely as a result at least in part of engagement of the newly functioning immune system.

This study has several limitations. Most significantly, our sample size was quite small. In addition, clinical considerations often led to inconsistent sampling based on patient and clinician availability. These considerations limit our ability to draw broad conclusions.

Opportunities to study similar systems of immune system/microbiome dynamics are present in SCID patients who undergo hematopoietic stem cell transplantation (HSCT). A pilot study investigated microbiome development in SCID patients after HSCT [55], though with 16S gene tag sequencing rather than shotgun sequencing, no healthy controls, a shorter sampling period, and no concurrent TCR sequencing. They found inconsistent results in microbiome diversity after transplantation, possibly due to the limited temporal range of the study, but did note that there were clear differences in the microbiota before and after transplantation in the four subjects studied.

## Conclusions

This study illustrates some of the uses of multi-omic data in assessing outcome in human gene therapy, including specifying aspects of T cell development and normalization of the microbiota with restoration of T cell function. As more of these studies are carried out, it will be possible to assess more fully the utility of such data. Of particular interest will be any signatures that help forecast outcome and provide new opportunities for initiating therapeutic interventions.

## Additional files

Additional file 1: **Table S1.** Patients studied. **Table S2.** Integration site sequencing statistics. **Table S3.** TCR-beta sequencing statistics. **Table S4.** Microbiome sequencing data. **Table S5.** Infections during timepoints analyzed in this study. (XLSX 38 kb)

Additional file 2: **Figure S1.** Stacked bar graphs summarizing genes at or near integration sites in each sample studied here. Integration site abundance was determined using the SonicAbundance method. The most abundant ten clones for each set are marked by the color code on the right. Gray indicates low abundance integration sites. Beneath each figure is indicated the time after cell infusion at which the sample was taken, and the number of total unique integration sites detected. **Figure S2.** Stacked bar graphs summarizing the TCRB analysis of PBMC samples. **Figure S3.** Bray-Curtis dissimilarity measures for the TCRB samples analyzed from PBMC. **Figure S4.** Population size estimates for progenitors and daughter T cells, with the minimum numbers of divisions required marked. Figure S5. Mean read counts for the metagenomic samples studied here. (PDF 959 kb)

## Abbreviations

EF1a: Elongation factor 1 alpha
HSC: Hematopoietic stem cell
HSCT: Hematopoietic stem cell transplantation
IL2RG: Interleukin 2 receptor subunit gamma
LTR: Long terminal repeats
MLV: Murine leukemia virus
SCIDn1: First SCID gene therapy trial
SCIDn2: Second SCID gene therapy trial
SCID-X1: X-linked severe combined immunodeficiency
TCR: T cell receptor
