# Supplementary material for: T cell dynamics and response of the microbiota after gene therapy to treat X-linked severe combined immunodeficiency
Source: Genome Med. 2018 Sep 28;10:70. doi: 10.1186/s13073-018-0580-z (PMC6161392; doi:10.1186/s13073-018-0580-z)
Supplement: Supplementary file 2 — Figure S1. Stacked bar graphs summarizing genes at or near integration sites in each sample studied here. Integration site abundance was determined using the SonicAbundance method. The most abundant ten clones for each set are marked by the color code on the right. Gray indicates low abundance integration sites. Beneath each figure is indicated the time after cell infusion at which the sample was taken, and the number of total unique integration sites detected. Figure S2. Stacked bar graphs summarizing the TCRB analysis of PBMC samples. Figure S3. Bray-Curtis dissimilarity measures for the TCRB samples analyzed from PBMC. Figure S4. Population size estimates for progenitors and daughter T cells, with the minimum numbers of divisions required marked. Figure S5. Mean read counts for the metagenomic samples studied here. (PDF 959 kb) [file 13073_2018_580_MOESM2_ESM.pdf]

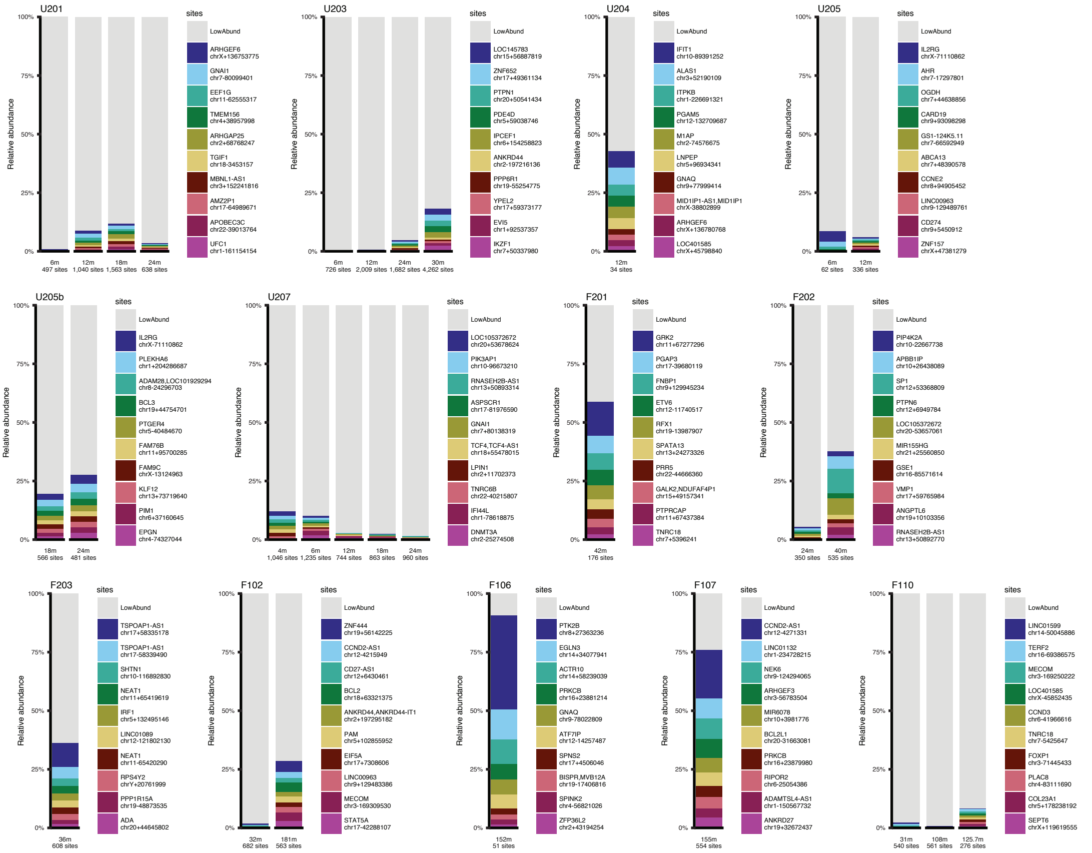

Figure S1. Stacked bar graphs summarizing genes at or near integration sites in each sample studied here.

Integration site abundance was determined using the SonicAbundance method. The most abundant ten clones for each set are marked by the color code on the right. Grey indicates low abundance integration sites. Beneath each figure is indicated the time after cell infusion at which the sample was taken, and the number of total unique integration sites detected.

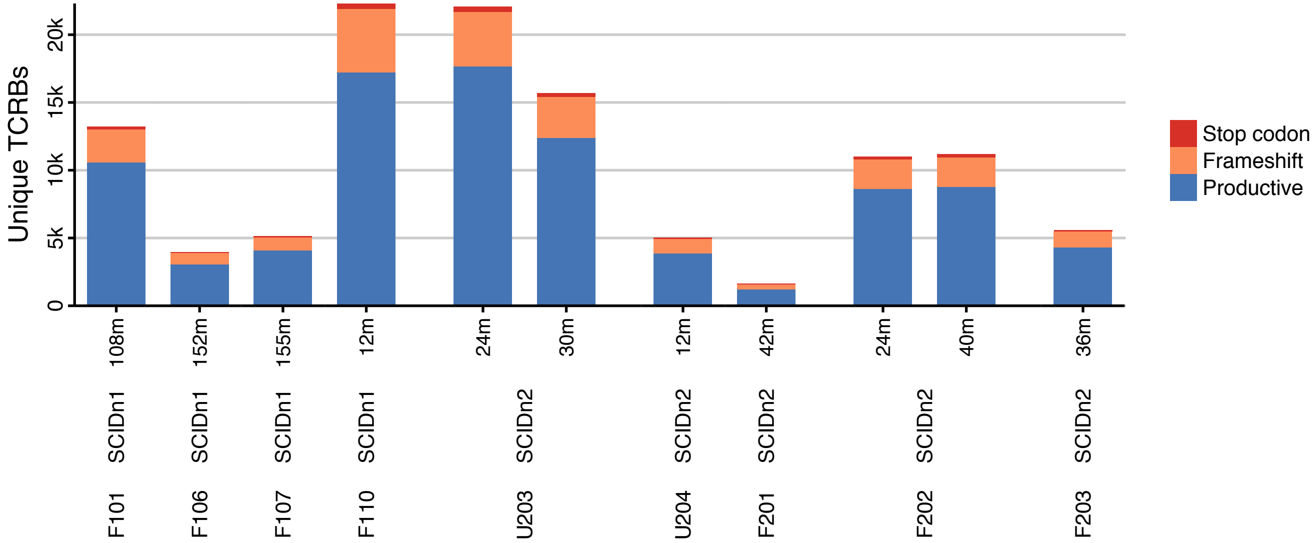

Figure S2. Stacked bar graphs summarizing the TCRB analysis of PBMC samples.

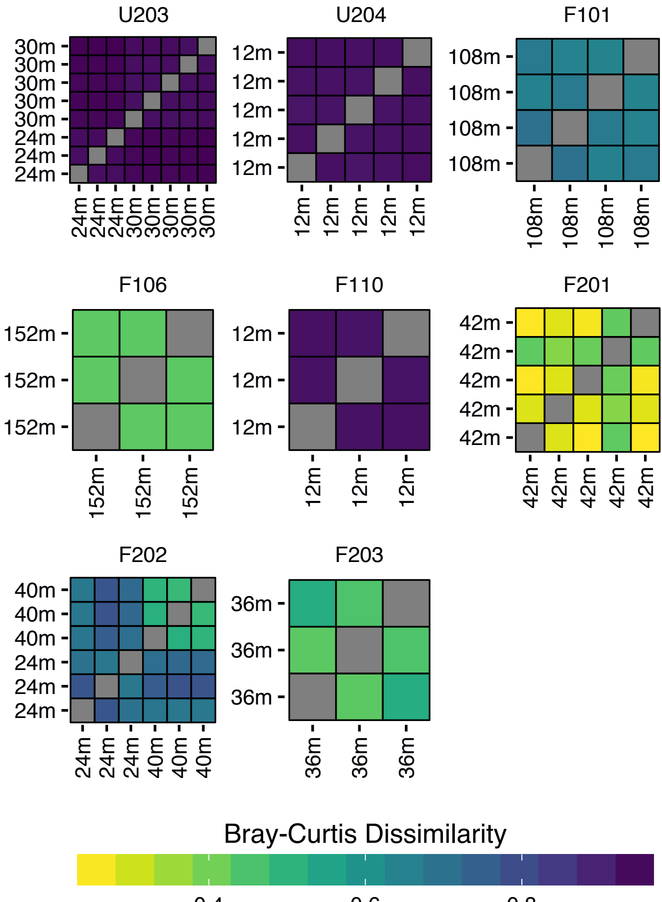

Figure S3. Bray-Curtis dissimilarity measures for the TCRB samples analyzed from PBMC.

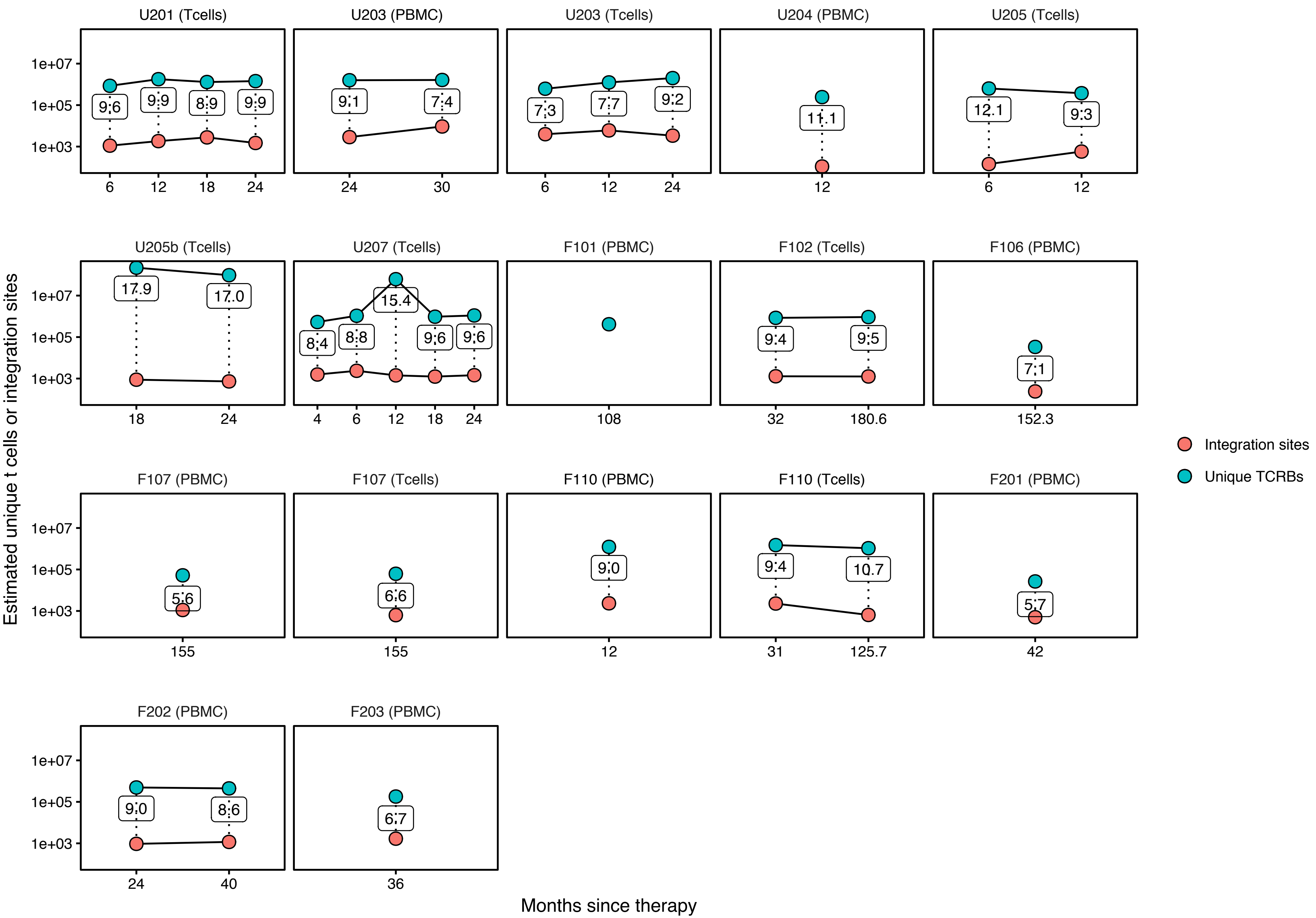

Figure S4. Population size estimates for progenitors and daughter T cells, with the minimum numbers of divisions required marked.

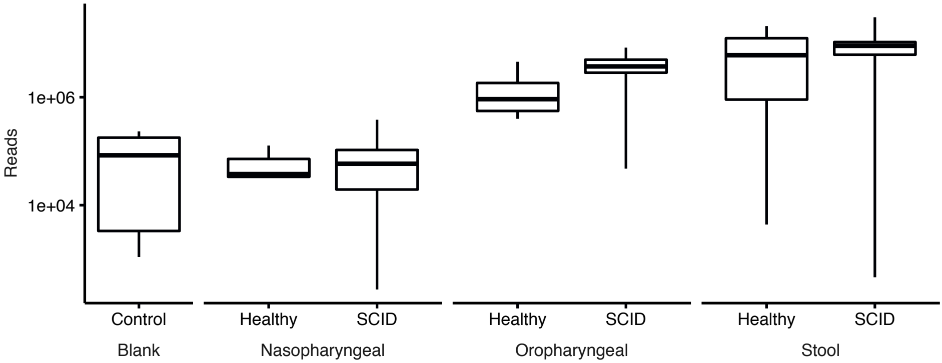

Figure S5. Mean read counts for the metagenomic samples studied here.
